# Traumatic hand injury management and outcomes: A case report

**DOI:** 10.4102/safp.v64i1.5479

**Published:** 2022-04-19

**Authors:** Monique M. Keller, Pieter W. Jordaan

**Affiliations:** 1 Department of Physiotherapy, Faculty of Health Sciences, University of the Witwatersrand, Johannesburg, South Africa; 2 Garden Route Hand Unit, Private Practice, George, South Africa

**Keywords:** hand injuries, surgeon, hand, occupational therapy, COVID-19

## Abstract

Acute hand injuries are routinely managed by family medicine and primary care physicians. An appropriate initial assessment and treatment, early referral to a hand surgeon when indicated, and timeous referral to a hand therapist are imperative. A patient case report is presented reporting on the initial and subsequent assessment, treatment and outcomes at 3, 6, 7 and 9 months for a patient who sustained an acute finger injury. Finger range of motion (ROM), sensation, pain, time of wound closure, hand function measured with the standardised disability of the shoulder, arm and hand (DASH) questionnaire were the outcomes used. Pain, crepitus, decreased sensation, decreased ROM right index finger proximal interphalangeal joint (PIPJ) and dense scarring was measured at 9 months. Missed injuries or lack of recognition of injury severity leads to delayed referral to specialist hand surgeons and therapists, which lengthens recovery time and leads to sub-optimal outcomes. This article aims to provide the primary care practitioner with the initial management of a patient who sustained a traumatic hand injury whilst using a power tool.

## Background

Research on the leading cause of hand injuries sustained during the COVID-19 pandemic in the United Kingdom showed an increase in injuries related to using machinery.^1^ Primary care physicians routinely manage patients after sustaining acute hand injuries but timeous referral to hand surgeons when the extent of the injury necessitates further assessment and management is crucial.^2^ The initial assessment, history taking and physical assessment are vital. It sets the scene for a complete objective hand assessment to ensure that all the compromised structures in the hand are optimally treated or a referral is made to a hand surgeon. A thorough history should include questions about the mechanism of injury, time of injury, hand dominance, the patient’s tetanus status and the baseline function and occupation of the patient.^2^ The initial physical assessment must include but are not limited to a thorough inspection of the injured hand, including a comparison to the contralateral hand, noting any abnormal hand posturing of the hand, fingers and wound position, shape and whether the wound is clean or contaminated. A neurovascular assessment including the capillary refill test, sensory testing and moving two-point discrimination test, active and passive range of motion (ROM), where indicated according to the observed injury, joint stability testing, must be performed.^2,3^

The management of acute hand injuries should be guided by the initial assessment and the wound. Open wounds must be irrigated and debrided.^2^ Primary wound management determines the patient’s functional outcome following a hand injury.^4^ Minimising oedema is imperative to hand injury management achieved through elevation^4^ of the affected hand above the heart level. Fractures can easily be missed in the absence of X-rays. Scar formation and joint stiffness are frequent complications following hand fractures.^4^ According to Karunadasa,^4^ splinting in the emergency room is helpful to assist in pain management following hand injuries.

Following traumatic hand injuries, the correct initial management is imperative to prevent substantial morbidity related to poor hand function, decreased quality of life and low work productivity. Timeous referral to a hand surgeon, a hand therapist, occupational therapists (OT) or physiotherapist, is of great importance to ensure optimal outcomes. The article aims to provide the primary care practitioner with the initial management of a traumatic hand injury patient sustained whilst using a power tool.

## Case presentation

**FIGURE 1:**
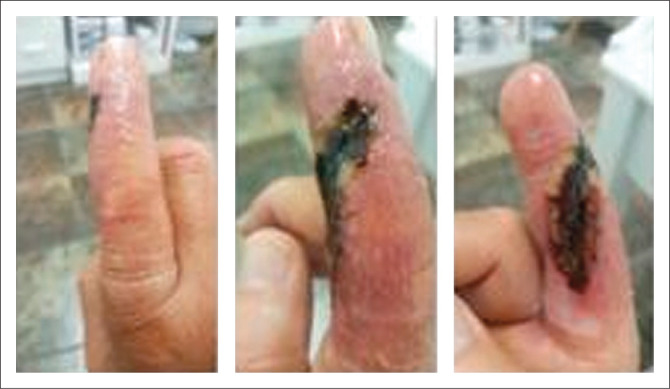
Index finger injury.

A 56-year-old right-hand dominant male sustained an index finger injury on 6 June 2020 whilst at home in South Africa during the national lockdown period. He works as a site manager, is a handyman and is an avid fisherman. The injury occurred at home whilst cutting a piece of wood with a small angle grinder fitted with a steel wood cutting blade. He lost control of the grinder and the blade cut into the radial side of his right index finger (Figure 1). He is a non-smoker has a background history of hypercholesterolemia with nil known drug allergies.

## Case management

The initial management included washing and suturing the wound by a primary care physician. After 13 days, the sutures were removed and the patient’s finger was passively flexed by the primary care physician with gaping of the wound observed by the patient. A wound care nurse was consulted every three days. Still, the patient became increasingly concerned about the wound and persistent pain. He consulted a hand surgeon on 2 July 2020, with the assessment presented in Table 1. The closest hand surgeon to the patient is approximately 130 km away.

**TABLE 1:** Preoperative hand surgeon’s assessments.

Outcomes	02 July 2020	27 August 2020
Wound observation	Open, dry, non-infected wound from the PIPJ, midline, volar to the radial side of the DIPJ	Nearly healed
Oedema	General presence	General presence
Tendons	Flexor digitorum profundus (FDP) intact. Flexor digitorum superficialis (FDS) unable to test.	Poor FDP glide
Range of motion	Active and passive severely limited	Severely decreased. Wound care specialist advised the patient to not move his finger too much, as she felt this hampered wound healing. Patient followed her orders.
Sensation	Ulnar digital nerve (UDN) normal. Absent radial digital nerve (RDN)	Radial digital nerve was 3/10 with 2-point discrimination (2-PD) 15 mm and monofilaments > 4 g.
Pain	Present with hypersensitivity	Present with hypersensitivity
X-ray	Taken 2 weeks post-injury: Middle phalanx radial aspect cortical disruption. Nondisplaced longitudinal fracture through distal phalanx, extending into the DIPJ	-

PIPJ, proximal interphalangeal joint; DIPJ, distal interphalangeal joint.

At 4 weeks after the date of injury, there was already a high likelihood of requiring a nerve graft, and therefore the decision was made to allow the wound to heal before doing the exploration and nerve graft. The wound care was performed by a local wound care nurse. After three months, wound closure was achieved. In the meantime, he was referred to an OT to regain ROM before surgery. No prior hand therapy referral was made. The patient’s primary complaints were hypersensitivity over the scar and the lack of finger ROM in the index and middle fingers.

## Investigations

As an adjunct to the initial assessment, an X-ray should have been taken at the first presentation, which would have identified the fracture earlier, instead of only being performed after two weeks.

## Differential diagnosis

The diagnosis made by the primary care physician was an uncomplicated laceration requiring wound care. The differential diagnosis for such an injury with an open wound should include assessment for possible digital nerve injury, flexor tendon injury and phalangeal fractures.

## Treatment

**FIGURE 2:**
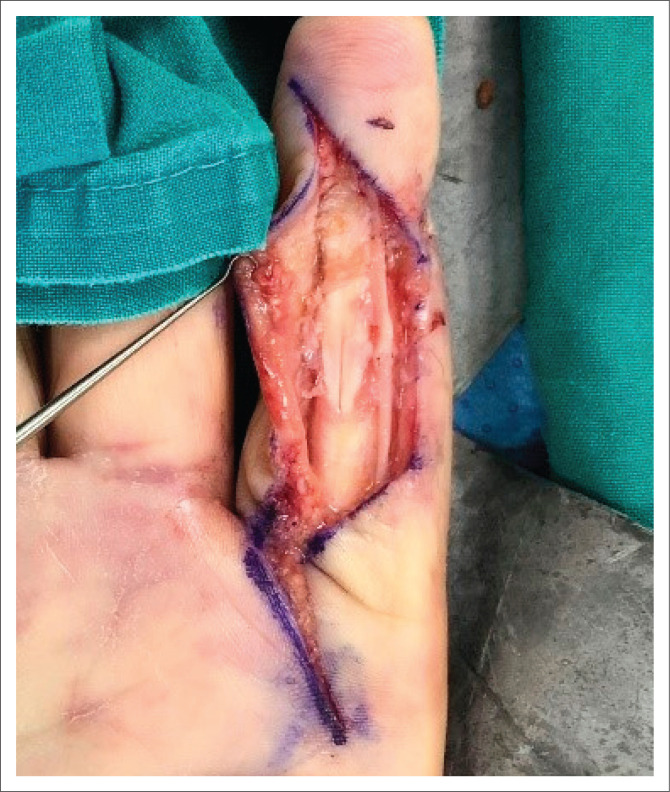
Posterior interosseous nerve graft of the radial digital nerve index finger.

Surgery was performed because of persistent symptoms of numbness on the lateral side of the index finger from the distal interphalangeal joint (DIP), hypersensitivity over the scar and loss of digital ROM. During surgery on 27 August 2021, the flexor digitorum profundus (FDP) tendon was found to be stuck in dense scarring and a thorough tenolysis was performed. He had a complete radial digital nerve (RDN) injury with a nerve gap repaired with a posterior interosseus nerve (PIN) autograft (Figure 2). He was placed in a bulky dressing and immediate active range of motion (AROM) was encouraged with referral for occupational hand therapy one week after surgery.

Throughout five sessions, OT treatment after the surgery included scar massage, desensitisation and oedema management through pressure sleeves. Active and passive joint mobilisation techniques were utilised, including a night extension splint and shotgun sleeve. A home exercise programme was prescribed, which he followed diligently.

## Outcome and follow-up

### Pain

The patient experienced pain and hypersensitivity on the palmar aspect of the index finger. At three months post-injury, he had pain of 8/10 on the visual analogue scale (VAS) over the scar. After five OT sessions, his pain and hypersensitivity improved, with increased hand function. At the 1-year follow-up, the patient complained of 3/10 (VAS) pain over the index distal interphalangeal joint (DIPJ) with movement of the index finger, but no pain at rest.

### Range of motion

Initial OT assessment revealed active ROM for the right index finger DIP 0° – 5° and PIP 30° – 60 (wrist active ROM of 45° extension and 60° flexion). After five sessions, he achieved active ROM of PIP 0° – 70° and DIP 0° – 35° (active ROM of the wrist extension: 50° and flexion: 70°).

### Function

The patient’s occupation involved working with tools. As a result of the hypersensitivity and decrease in ROM, he struggled to perform his duties at work. He also struggled with self-care such as fastening buttons. Hand function was assessed with the disability of the shoulder, arm and hand (DASH) questionnaire, where a possible score is out of 100. A score of 100 indicates extreme disability, and 0 shows a fully functional patient with no disability related to the arm, shoulder or hand injury. At three months post-injury, a DASH score of 72.5 and at six months post-injury, a DASH score of 53.33 were calculated.

### Follow-up

At follow up on 30 March 2021, approximately seven months after surgery and nine months after his injury, he was satisfied with his recovery but developed new pain in his index finger DIPJ. Clinically he had crepitus, which is likely post-traumatic osteoarthritis because of the injury. His ROM of his right index finger proximal interphalangeal joint (PIPJ) was 30° – 94° with a fixed flexion deformity of 30° and his DIPJ 10° – 30°. There were still areas of dense scarring. He had a 2 PD of 10 mm and monofilaments of 2.0 g of his RDN, compared with 4 mm and 0.5 g for his ulnar digital nerve (UDN). His ROM was functional and he had recovered protective sensation.

## Discussion

The case report patient did not have immediate access to a specialised hand surgeon with delayed recognition and incorrect management leading to long-term consequences for a patient’s quality of life, function and work.^2,4^ Serious hand injuries with chronic pain correlate with low quality of life, anxiety and depression.^5^

Primary care physicians routinely manage patients who sustained traumatic hand injuries but a thorough assessment and subsequent referral to a hand surgeon is imperative in complex hand injuries.^2^ Karunadasa identified neurological assessment and two views X-rays, as routine assessments, and fundamental care principles for hand injury management.^4^ In this patient’s case, an RDN injury, an undisplaced longitudinal fracture through the distal phalanx and a stabile cortical middle phalanx fracture were missed in the initial assessment. Early nerve repair is essential^4^ and a primary nerve repair carried out by a hand surgeon could have improved the sensory outcome, possibly avoiding the need for a nerve graft and decreasing the pain relating to the RDN injury. It is important to remember that sensation plays a more significant part in the hand compared with other parts of the body.^3^

We can only speculate about the factors contributing to the delay in referral. The COVID-19 pandemic has definitely caused a delay in presentation to emergency departments and a long distance to travel has also been observed as a cause for delay in presentation.^6^ In this patient, both reasons are relevant. A failure to recognise the severity of the soft tissue injury, the nerve injury and the open fracture likely contributed to this as the initial thought would have been that the injury was not severe enough to warrant referral, risk exposure to COVID-19 and incur the cost of travel. However, early and skilled treatment is essential in ensuring better outcomes^2,4^ and therefore early referral of these patients is crucial.

## Teaching points

- Power tools cause deep and complex injuries to multiple structures.
- Determining whether a tendon injury occurred is imperative through assessing the flexor digitorum superficialis (FDS) by blocking all fingers except the injured finger into extension and then asking the patient to flex the PIPJ of the affected finger. Assess the FDP by blocking the PIPJ and asking the patient to flex the DIP. No movement present indicates a potential injury. Observation is vital in assessing a possible tendon injury as the hand loses the natural cascade position. To evaluate a potential extensor tendon injury, ask the patient to extend the involved finger while blocking the proximal joints. It is important to note that it can be very difficult to adequately assess tendon function in the presence of an underlying fracture.
- When presented with a patient who sustained an acute traumatic hand injury, do a thorough hand assessment (especially neurovascular) and two views X-rays.
- With complicated hand fractures, neurological or vascular fall out, tendon involvement or complicating wound and soft tissue injuries, refer the patient to a hand surgeon without delay. If in doubt, contact your local hand surgeon to discuss the case.
- The role of the primary care physician is to provide adequate pain control, irrigate the wound and in so doing remove any gross contamination. Control active bleeding with direct compression and loosely approximate the skin as far as possible. Depending on the experience and available facilities, the primary carer should use local anaesthetic for further examination and wound irrigation at the time of skin approximation, thus influencing the need for specialist referral.
- Early referral of the patient to a qualified OT or physiotherapy with a special interest (or advanced training) in hand injuries will assist with oedema management and ROM.
- Nerve injuries of the fingers are repairable.
